# Factors Affecting Early Antibody Secreting Cell Maturation Into Long-Lived Plasma Cells

**DOI:** 10.3389/fimmu.2019.02138

**Published:** 2019-09-11

**Authors:** Doan C. Nguyen, Chester J. Joyner, Iñaki Sanz, F. Eun-Hyung Lee

**Affiliations:** ^1^Division of Pulmonary, Allergy, Critical Care, and Sleep Medicine, Department of Medicine, Emory University, Atlanta, GA, United States; ^2^Yerkes National Primate Research Center, Emory University, Atlanta, GA, United States; ^3^Division of Rheumatology, Department of Medicine, Emory University, Atlanta, GA, United States; ^4^Lowance Center for Human Immunology, Emory University, Atlanta, GA, United States

**Keywords:** immunoglobulin, B cell, antibody-secreting cell, long-lived plasma cell, differentiation, maturation, maintenance

## Abstract

Antibody secreting cells (ASCs) are terminally differentiated cells of the humoral immune response and must adapt morphologically, transcriptionally, and metabolically to maintain high-rates of antibody (Ab) secretion. ASCs differentiate from activated B cells in lymph nodes and transiently circulate in the blood. Most of the circulating ASCs undergo apoptosis, but a small fraction of early ASCs migrate to the bone marrow (BM) and eventually mature into long-lived plasma cells (LLPCs). LLPC survival is controlled both intrinsically and extrinsically. Their differentiation and maintenance programs are governed by many intrinsic mechanisms involving anti-apoptosis, autophagy, and metabolism. The extrinsic factors involved in LLPC generation include BM stromal cells, cytokines, and chemokines, such as APRIL, IL-6, and CXCL12. In humans, the BM CD19^−^CD38^hi^CD138^+^ ASC subset is the main repository of LLPCs, and our recent development of an *in vitro* BM mimic provides essential tools to study environmental cues that support LLPC survival and the critical molecular mechanisms of maturation from early minted blood ASCs to LLPCs. In this review, we summarize the evidence of LLPC generation and maintenance and provide novel paradigms of LLPC maturation.

## Introduction

**Figure 1:**
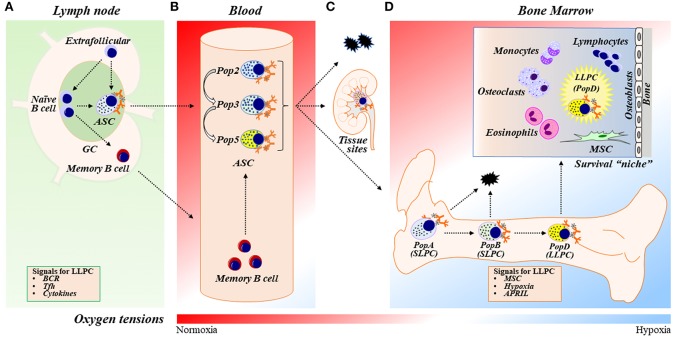
ASC differentiation, maturation, and LLPC generation and maintenance. **(A)** In response to vaccination or infection, naïve B cells proliferate and differentiate into ASCs within germinal center (GC) reactions in secondary lymphoid organs (SLOs) such as lymph nodes (LNs) or outside of GCs in extrafollicular responses. ASCs then egress out of SLOs and circulate in the blood transiently. Naïve B cells can also differentiate into memory B cells, circulate systemically, and further differentiate into ASCs upon activation. The two-step model of LLPC generation consists of the GC reaction and the BM maturation process [see also **(D)**]. In the GC reaction, B cells may receive signals such as BCR stimulation, Tfh (or other Th cell) interactions, and cytokines (such as IL-21 and IFNγ) in the GC that are important during ASC differentiation to ultimately become a LLPC. **(B)** Human blood ASCs are heterogeneous and can be classified into distinct populations (Pops), including Pop2, Pop3, and Pop5, based on their surface protein expression (see also Table 1). **(C)** Most blood ASCs apoptose. However, a fraction migrates to inflammatory or other tissue “niches” (e.g., mucosa and bone marrow) and become tissue-specific ASCs. **(D)** A small number of blood ASCs emigrate to the BM, a physiologically hypoxic environment. Human BM ASCs can be identified based on surface protein expression as PopA (SLPCs), PopB (SLPCs), and PopD (LLPCs), respectively, in the BM (Table 1). LLPCs acquire longevity and are maintained within the dedicated BM survival “niche.” ASC survival factors include BM MSCs (and/or their secreted factors), cytokines (i.e., APRIL), and hypoxic conditions. BM, bone marrow; GC, germinal center; ASC, antibody-secreting cells; SLPC, short-lived plasma cells; LLPC, long-lived plasma cells; BCR, B-cell receptor; Tfh, T follicular helper cells; MSC, mesenchymal stromal/stem cells.

A key aspect of the adaptive immune response is the rapid production of high-affinity antibodies (Abs). This antibody production is the function of an antibody secreting cell (ASC), which arises from naïve (or memory) B cells as they encounter antigen, activate, proliferate, and differentiate. In draining lymph nodes, the B cell to ASC differentiation can occur either in the germinal center (GC) reactions (which has implications for ASC longevity; see below) or outside GC, as a part of an extrafollicular response (1–3) (Figure 1A). Differentiated ASCs subsequently egress out of lymph nodes and circulate in the blood (Figure 1B). Despite their critical function, ASCs are rare and comprise of no more than ~0.01–1% of the total cellularity in the circulation and lymphoid tissues.

In the blood and secondary lymph organs (SLOs), the ASC presence is transient (days to a few weeks) during primary and secondary immune responses (4–7). After vaccination or during infection, ASCs appear in circulation for days and quickly disappear from the blood while serum Ab titers remain (6, 8–10). The bimodal characteristics of serum Ab decay after infection or long-lived vaccines suggests that the large initial burst of ASCs is responsible for the peak of serum Ab titers and the secondary decay is due to ASCs with longer half-lives (11).

**Table 1:** Phenotype of blood and BM ASC subsets^*^.

**ASC subsets**	**Blood**	**Pop2**	**Pop3**	**Pop5**
	**BM**	**PopA (SLPC)**	**PopB (SLPC)**	**PopD (LLPC)**
FACS markers	CD19	**+**		neg
	CD138	neg	**+**	
	CD38	**++**		

* *BM, bone marrow; ASC, antibody-secreting cell; SLPC & LLPC, short- & long-lived plasma cell; neg, negative*.

*Color represents similar surface markers*.

In the circulation, the majority of circulating ASCs undergo apoptosis; yet a specialized fraction further matures into long-lived plasma cells (LLPCs) as they migrate to BM or other tissues (12) (Figures 1C,D). LLPCs are quiescent, terminally-differentiated, non-dividing cells that survive after the antigen vanishes and is responsible for protection (4, 13–15). It is thought that LLPCs constitutively produce specific Abs for years or even decades after an initial infection or immunization (12), with evidence that they can persist independently of memory B cells (11, 15–17). Hence, generating LLPCs is the main objective of an ideal vaccine.

Many studies show that ASCs derived from GC reactions, rather than extrafollicular responses, have the potential for LLPC formation and survival (12, 18–21). After B cell receptor (BCR) cross-linking and costimulation, a number of cytokines (particularly IL-21 and IFNγ), through help from T follicular helper cells (Tfh), and affinity maturation have significant intrinsic influence on an ASC generated through GC responses to become a LLPC (20–28). Most recently, IFNγ through B cell intrinsic T-bet expression is required for LLPC generation (29). Thus, the GC appears to be important for the formation of LLPCs, yet the overall molecular mechanisms and programs that govern these processes are largely unexplored.

Some investigators show that LLPCs can be generated independently of B cell maturation (i.e., in the absence of GC formation) (30–32). However, these studies showed persistence of ASCs for only 3–4 months and it is unclear if they are indeed long-lived. Nonetheless, whether a memory B cell generated initially through a GC response and re-stimulated extrafollicularly has similar intrinsic LLPC potential is not entirely known. Thus, it is still debated if some ASCs derived extrafollicularly from memory vs. naïve B cell origins may have long-lived survival potential.

In this review, we focus on the molecular and cellular processes involved in the adaptation of ASCs after exiting the GC into the medullary cords and efferent lymphatics and upon entering the blood, and eventually the BM niche (Figure 1D). Throughout the review, we will refer to “differentiation” as the process in which a B cell becomes an ASC (through GC or non-GC reactions), and “maturation” as the process in which an early minted ASC develops or transforms into a LLPC. The term “maintenance” will be used for survival of the ASCs. On this basis, we will discuss the role of BM “microniches” in both the maturation and the maintenance of BM LLPCs.

## Transformation of B Cells to ASCs and Then to LLPCs

The prime directive of ASCs is to synthesize and secrete Abs that provide protection against microorganisms. ASCs produce large amounts of Abs, typically at a rate of between ~10 and 1,000 (and can be up to ~5,000–10,000) Ab molecules per cell per second, which corresponds to ~0.2–22 pg/cell/day (33–38). To perform this heroic feat, ASCs undergo fundamental changes in morphology and homeostasis during terminal differentiation, which include altering cellular structure, surface protein expression profile, metabolism, and other cellular and molecular programs. In this section, we discuss these changes by contrasting the recent findings of human blood and BM ASCs.

### Cellular Structure

**Figure 2:**
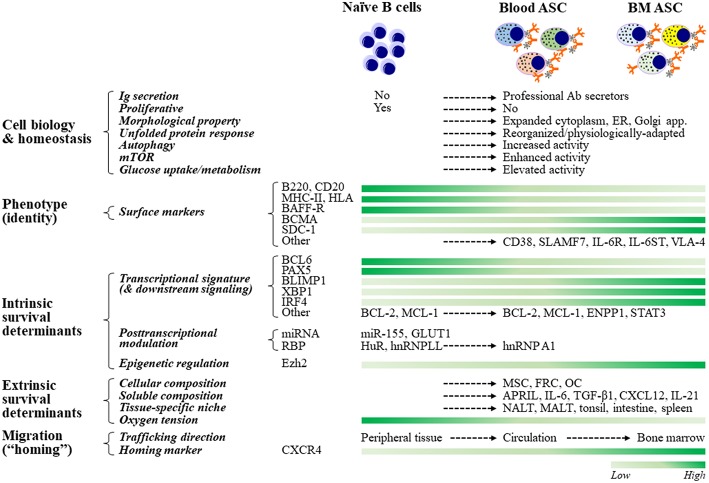
ASC cell biology, fate determination, and resultant phenotypes. ASCs adapt their cellular biology, machinery, and morphology to maintain homeostasis while producing copious amounts of antibodies. For example, ASCs must upregulate the unfolded protein response (UPR) to inhibit apoptotic pathways that would be engaged due to increased Ig synthesis. Other pathways that act in concert with the UPR to support ASC homeostasis are autophagy, mTOR, and glucose metabolism. Although no phenotype-based “universal identity” of blood and bone marrow ASCs presently exists, ASCs are typically identified by increased expression of CD38, CD138, and BCMA, and decreased expression of B220, MHCII, and BCR. The commitment to the ASC fate of naïve B cells is regulated transcriptionally, post-transcriptionally, and epigenetically. Maintenance of B cell programs is mainly controlled by Bcl6 and Pax5, and promotion of B cell differentiation and ASC commitment are essentially governed by Blimp1 (encoded by *Prdm1*), Xbp1, and Irf4. The role of post-transcriptional (through certain miRNAs and RBPs) and epigenetic (such as via *Ezh2*) regulation is increasingly being recognized. The lifespan of ASCs ranges from a few days to several decades and is thought to be chiefly determined extrinsically, which is largely dependent on their residency. Accordingly, LLPCs reside in a specialized, dedicated BM compartment (“survival niche”) that is physiologically hypoxic and is formed and maintained by both cellular (such as MSCs, FRCs, and OCs) and soluble (such as APRIL, IL-6, TGF-β1, and CXCL12) components. Other tissue-specific niches capable of maintaining ASCs have also been described. ASC migration (or “homing”) from peripheral tissues or SLOs toward survival niches is primarily directed by binding of CXCR4 to its ligand, MSC-secreted chemokine CXCL12 (SDF1α). Certain miRNAs and RBPs include miR-155, which is involved in cytokine production, and HuR and hnRNPLL, which modulate IgH mRNA turnover and translation. ASC, antibody-secreting cells; LLPC, long-lived plasma cells; ER, endoplasmic reticulum; SLO, secondary lymphoid organs; MSC, mesenchymal stromal cells; FRC, fibroblastic reticular cells; OC, osteoclasts; NALT, nasal-associated lymphoid tissues; MALT, mucosa-associated lymphoid tissues.

To accommodate the production of copious amounts of proteins, ASCs expand their cytoplasm, endoplasmic reticulum (ER), and Golgi apparatus during differentiation and maturation, enabling increased capacity for Ab synthesis and secretion (39) (Figure 2). Early ASCs lose their proliferative capacity and reduce the nucleus size by condensing their chromatin. The decrease in nuclear area and the repositioning of the nucleus closer to the cell membrane enable ASCs to accommodate additional cellular machinery necessary for protein production. Overall, these morphological changes are orchestrated to result in assembling an Ab factory.

### Blood ASC Phenotypes

As ASCs differentiate and mature, they alter their surface protein expression profile. These proteins can be used as the phenotypic markers for identifying subsets within the ASC population. Notably, there is no universally accepted or “standardized” phenotype to identify ASCs—in humans or in mice (which make it difficult to compare across studies). Nevertheless, human ASCs in general lose their surface immunoglobulins and can typically be identified based on the expression of one or more of the following cell surface markers: CD19, CD27, CD38, CD138, and BCMA (40–46) (Figure 2). In mice, blood ASCs typically reduce or lose the expression of B220, MHC-II, and BCR. Additional markers such as Sca-1, TACI, and CD98, have also recently been used for identifying ASCs in mice—on the basis of CD138 expression (43, 45–49); however, it is unclear how this combination translates to humans or other animal models.

In humans, blood ASCs are typically identified as CD19^int^CD27^hi^CD38^hi^ (gated on CD3^−^CD14^−^IgD^−^ cells) (41). However, our recent studies along with others have demonstrated that human blood ASCs are heterogeneous and can be further subgrouped on the basis of the additional expression of CD19 and CD138 (40, 50) (Figures 1B, 2, Table 1). Of the five different ASC subsets we identified, three were CD19^+^ whereas two were CD19^−^ subsets. Among them, populations 2 (pop 2; CD19^+^IgD^−^CD27^+^CD38^hi^CD138^−^) and 3 (pop 3; CD19^+^IgD^−^CD27^+^CD38^hi^CD138^+^) are the most abundant, and population 5 (pop 5; CD19^−^IgD^−^CD27^+^CD38^hi^CD138^+^) resembles LLPCs by surface markers (Table 1). The other ASC subsets contained a mixed population of B cells and other cell types and thus, the study focused on pops 2, 3 (CD19^+^), and 5 (CD19^−^). Based on RNAseq analysis, pops 2 and 3 are very similar transcriptionally whereas pop 5 is quite distinct (Figure 1B). More interestingly, all pops 2, 3, and 5 have similar potential for survival in an *in vitro* BM mimetic system, suggesting the importance of extrinsic factors in their survival.

### Blood ASC Migration

The exit of ASCs from SLOs into the blood is guided by the expression of their S1P receptor 1 (S1P1) (51, 52). Subsequently, in concert with changes in surface markers, blood ASCs modulate the expression of chemokine receptors that direct them to local tissues (Figure 1C). For example, CXCR3 directs ASCs to inflamed tissues, CCR9 guides them to the small intestine, CCR10 directs the cells to a variety of tissues, including the colon, lung, trachea, or mammary glands (53), and, importantly, CXCR4 leads them to the BM rich in CXCL12 (54–56). It is also thought that ASCs are retained in the BM by CXCR4 because they fail to accumulate in the BM microenvironment in CXCR4^−/−^ mice (54).

It still remains unclear how the expression of chemokine receptors on ASCs is regulated, although transcription factor c-Myb may be involved (57). In the absence of c-Myb, no ASCs are detected in the BM. Whether this phenomenon is due to inability of c-Myb deficient ASCs to migrate along a CXCL12 gradient or if c-Myb is involved in ASC survival will need further elucidation. Nonetheless, the migratory capability of ASCs appears to be decreased as they mature (55, 58), along with upregulation of Blimp-1 (59–61). This phenomenon may serve as another means of retaining ASCs in the BM or other tissues. Clearly, migration to specific sites rich in survival factors enhances ASC survival; but whether fundamental intrinsic differences are imprinted during ASC differentiation or whether maintenance is solely dependent on homing markers alone is not well-established. Thus, the nature (intrinsic) or nurture (extrinsic) is a critical debate for LLPC generation. To further its complexity, ASC maturation to a LLPC may actually require a combination of both.

### BM LLPC Phenotype

As previously mentioned, LLPCs are non-dividing, long-lasting terminally differentiated ASCs. In mice and humans, most LLPCs reside in the BM (12, 62, 63) (Figure 1D). These specialized ASCs persist and are thought to autonomously and continuously produce Abs for decades or beyond, independently of antigen stimulation (13–15). BM ASCs are also thought to be unresponsive to further antigenic stimulation (64). However, unlike IgG ASCs, a recent report identified a population of IgM BCR expressing ASCs that may sense antigen (65). In mice, ASCs in the spleen and the BM are identified based on the expression of B220, CD138, and Sca-1 subsets, with implications of B220^−^ ASCs as mature subsets (46–48). Human BM ASCs are heterogeneous, with few overlapping markers with mice, and can be grouped on the basis of expression of CD19, CD27, CD38, and CD138 (66) (Figure 1D, Table 1). To identify the human BM LLPC subset, we interrogated each compartment for microbial specificity against viral infection (measles and mumps) that occurred decades ago and found that only the CD19^−^CD38^hi^CD138^+^ subset contained these long-lived viral specificities. We assigned the CD19^+^ BM populations as short-lived plasma cells (SLPCs), which include pops A and B that did not comprise of the long-lived specificities (66) (Table 1). Others have alluded to these results but did not show long-lived antigen specificities (67). Thus, it appears that the ASC fraction that downregulates CD19 expression and escapes apoptotic cell death eventually becomes LLPCs.

## Intrinsic Regulation of ASC Differentiation and Maintenance

The B cell to ASC differentiation involves an extensive reorganization of transcriptional networks (68, 69) (Figure 2). In order for ASCs to synthesize and secrete large amounts of Abs, these regulatory networks massively upregulate transcription of the heavy and light chain mRNA transcripts (up to 100–1,000-folds). ASCs have unique transcriptomes where >70% transcripts encode the IgH and IgL chains for Ab synthesis (49). The differentiation of ASCs is regulated at the transcriptional, post-translational, and epigenetic levels. In this section, we discuss the intrinsic cellular programs—including signaling pathways and metabolic alterations as well as transcriptional, post-transcriptional, and epigenetic networks—involved in regulation of ASC differentiation, maturation, and maintenance.

### Transcriptional Regulatory Networks

Aspects of ASC biology, metabolism, homeostasis, migration, and survival are controlled by their distinct gene expression programs (or transcriptional regulatory networks) (49, 70, 71). These regulatory programs promote features to produce large quantities of Abs and to shed B cell characteristics. They function through two groups of antagonistic transcription factors, in which acquisition of plasma cell-specific transcription results in termination of B cell-specific transcriptional program (39, 72–74).

Transcription factors maintaining the B cell program are mainly BCL6 and PAX5 (75), although Bach2 and IRF8 are also important (39, 69). BCL6 is highly expressed in GC B cells and promotes cell proliferation and survival. Transcription factors facilitating ASC differentiation include Blimp1 (encoded by *Prdm1*) (76), XBP1, and IRF4 (39, 60, 61, 69, 77–79). These ASC-“specific” transcription factors are uniquely upregulated in plasma cell transcriptome (49, 70), with Blimp1 and XBP1 required for Ab secretion whereas IRF4 is likely essential for survival (61, 80–82). Importantly, Blimp1 and XBP1 play a multifunctional role in both instructing differentiation and maintaining homeostasis. Blimp1 inactivation downregulates the unfolded protein response (UPR), thus negatively affecting the cell secretory capabilities (60, 61). Also, Blimp1 positively regulates mTORC1 activity (61) as loss of Raptor negatively affects ASC maturation and Ab secretion (but not survival) (48). Finally, XBP1 has been shown to be an important regulator of the UPR (83).

In addition to these two reciprocally-regulated groups of transcription factors, a number of other factors have been found differently expressed by ASCs in distinct compartments (i.e., SLOs, blood, and BM). Examples include anti-apoptotic factors, such as Mcl-1, Bcl-2, BclxL, Bim, and BCLw, which are increased, and pro-apoptotic factors, such as Bax and BID, which are decreased, to promote ASC maintenance (84–87). Importantly, expression of Mcl-1 appears to be required for ASC survival in the BM and is mediated by BCMA, a TNF receptor (85), potentially under the control of IRF4 (71) or Zbtb20 (61, 88–90).

### Signaling and Metabolism

During differentiation, ASCs adapt and utilize various signaling pathways to accommodate the need for high-rate Ab production and maintenance of homeostasis (Figure 2). Thus, activation of the UPR, the cellular response to ER stress due to accumulation of misfolded (damaged) proteins, is essential for increased Ab secretion during ASC differentiation from B cells (61, 81, 91, 92). In most cells, upregulation of the UPR indicates the inability of the cells to recycle proteins via ubiquitination or signify the hijacking of this pathway (i.e., by virus). In these situations, the UPR typically results in apoptosis as a means to eliminate a virally infected or dysfunctional cell. In the case of ASCs, the UPR is upregulated due to stress from the massive protein secretion in order to maintain the cell's survival since this imbalance could quickly lead to apoptosis. This pathway strongly involves XBP1 through ATF6 and IRE-1 during differentiation (61, 91, 92). Nevertheless, little is known about the regulation of this pathway in human ASCs after differentiation or how it may be modulated to ensure the maintenance of LLPCs. Thus, identifying the molecular mechanisms that regulate this pathway could lead to modulating LLPC formation and maintenance.

Antibody secreting cells (ASCs) must also adapt their metabolism to produce Abs during circadian nutrient fluctuations while maintaining cellular homeostasis. As terminally-differentiated cells, ASCs do not actively remodel their cytoplasm through cell division but rely on autophagy to recycle their protein aggregates and organelles in order to optimize energy for survival. Multiple autophagy genes, such as *Atg5, Atg9*, and *Atg13*, are upregulated in ASCs and likely play a role as LLPCs mature (49, 66, 93). This increased autophagic activity enables LLPCs to regulate the production of Abs when nutrients are limited (93). Interestingly, hypoxia is a known inducer of autophagocytic pathways, and we recently showed that hypoxic conditions enhance the survival of human ASCs *in vitro* (94). Whether autophagy programs are upregulated prior to BM localization or in response to the hypoxic BM microenvironment is still not clear.

Another important signaling pathway for ASCs is the mTOR. The mTOR kinase is a major regulator of many cellular processes, including survival and proliferation. mTOR signaling, mainly through the mTORC1 signaling complex (mTORC1), regulates the biosynthesis of cellular macromolecules, including proteins, nucleic acids, and fatty acids, as well as glycolysis and organelle biosynthesis (95, 96). Blimp1 positively regulates mTOR signaling as B cells differentiate into an ASC (48, 61, 97). However, in humans, as ASCs mature into BM LLPCs, mTORC1 activity is downregulated (94) and autophagy is increased (66). Additionally, the survival of human BM LLPCs, unlike that of BM SLPCs or early minted blood ASCs, is resistant to mTOR inhibitors, illustrating downregulation of mTOR pathways for LLPC maturation (94). Similar findings were corroborated in mouse studies (48).

As ASCs evolve into LLPCs, major metabolic changes occur. In addition to differences in mTOR signaling that functionally distinguishes LLPCs from SLPCs, a sundry of metabolic mechanisms differs between these two cell types. LLPCs have higher glucose uptake than SLPCs, and utilize glucose for Ab glycosylation (98). Moreover, LLPCs have increased expression of the glucose transporter, GLUT1 (98, 99). Recently, the ATP-degrading enzyme ENPP1, a regulator of glucose metabolism, was shown to be required for the development and survival of LLPCs in mice (100). In both mice and human, higher maximal respiratory capacity was shown in LLPCs compared to SLPCs (98), suggesting differences in respiratory capacity that may be linked to survival advantages. Nonetheless, more studies are warranted to understand whether versatility of energy utilization may determine survival advantages in unique nutrient-deprived environments.

### Post-transcriptional Modulation

Post-transcriptional regulation of mRNA expression is a major mechanism to rewire the transcriptome and proteome. This type of regulation defines the fate of mRNAs, including immunoglobulin transcripts, through multiple steps of mRNA processing, including alternative splicing of pre-mRNAs, mRNA stability and turnover, 3' UTR regulation, and translational control (101–103). The two key players in post-transcriptional regulation of protein expression are non-coding RNAs (mainly miRNAs) and associated RNA-binding proteins (RBPs) (101, 103, 104). In committed ASCs, post-transcriptional regulation is employed in several processes and can play a role in the magnitude of immunoglobulin expression (Figure 2). The processing of *IgH* mRNA is modulated post-transcriptionally (which requires Blimp-1) (60, 61). Expression of the glucose transporter GLUT1 is also regulated at the post-transcriptional level (98, 99). However, whether post-transcriptional regulation of mRNA and non-coding RNAs plays a role in LLPC maturation needs further evaluation.

RNA-binding proteins (RBPs) are involved in regulating the B cell programs and thus, can also influence ASC differentiation (101, 102). An example is HuR, an RBP splicing regulator ubiquitously found in the nucleus and capable of nucleo-cytoplasmic shuttling (105). Depletion of HuR results in unbalanced mitochondrial metabolism and impaired B cell proliferation and differentiation, which has a negative impact on ASC differentiation (106). Another major RBP involved in ASC differentiation is the splicing factor hnRNPLL, a member of the hnRNP (heterogeneous nuclear ribonucleoprotein) family. hnRNPLL is specifically induced in ASCs and, through regulating mRNA alternative splicing and stability, facilitates B cell differentiation and ASC Ab secretion (107). Also, hnRNPLL and the transcription elongation factor ELL2 (elongation factor, RNA polymerase II, 2), a regulator of pre-mRNA processing in plasma cells, modulate the ratio of secreted vs. membrane-encoding IgH transcripts. Most interesting, ELL2 was responsible for differentially processed transcripts such as BCMA (108).

Our recent integrated transcriptomic and proteomic analysis distinguishing early minted ASCs and BM LLPCs identified a novel RBP, hnRNP A1, as a potential post-transcriptional modulator (94). Blocking hnRNP A1 led to reduced survival of both human blood ASCs and BM LLPCs (109). Thus, unlike HuR or hnRNPLL, which are involved in regulating the differentiation of B cells to ASCs, hnRNP A1 may be unique and important in the regulation of the maturation of early blood ASCs and BM LLPCs. Altogether, these data highlight the important role of post-transcriptional regulation in the differentiation as well as maturation of ASCs, which can also influence the survival of LLPCs.

### Epigenetic Regulation

*Ezh2*, a histone methyltransferase that catalyzes trimethylation of histone H3 (H3K27me3), has been shown to epigenetically regulate gene expression during ASC differentiation. *Ezh2* expression is upregulated during ASC differentiation, and *Ezh2*-deficient ASCs exert reduced levels of the UPR, glucose metabolism, and mitochondrial respiration, resulting in decreased Ab secretion (110, 111) (Figure 2). Thus, *Ezh2* plays a role in ASC differentiation by promoting metabolic changes that are important for Ab secretion. Interestingly, this enzyme can interact directly with Blimp1 (60) and represses the B cell program (110). It will be important to understand if *Ezh2* expression is modified as ASCs mature into a LLPC.

## Extrinsic Factors in ASC Maturation and Maintenance

The notion that some ASC subsets exist for only a few days whereas others live for decades is thought to be chiefly determined extrinsically, i.e., largely dependent on where ASCs reside. In both mice and humans, most LLPCs reside in a specialized BM microenvironment, known as the survival niche (12, 62, 63) (Figure 1D). Removal of ASCs from this specialized residence leads to rapid cell death. Thus, these niches enable ASC maintenance and longevity—via intracellular crosstalk or direct ASC interactions with neighboring cells and/or through soluble factors produced locally by neighboring cells (12, 112, 113). It is believed that these niches support only a limited ASC number due to their physical confines (112). Thus, the BM environment must be stable to protect their resident cells yet dynamic enough to adapt to new arrivals.

Although LLPCs are highly enriched in the BM (63), recent studies have described several tissue-specific niches (TSNs) that afford LLPC survival advantages. LLPCs can also be found, albeit at low frequencies, in other tissues throughout the body, such as the nasal-associated lymphoid tissues (NALT) (114), human tonsillar lymphoid tissues (115), human mucosa or mucosa-associated lymphoid tissues (MALT) (116, 117), human intestine (118–120), spleen (67, 121), and human salivary gland microenvironment (in primary Sjögren's syndrome) (122) (Figure 2). In mice, the spleen appears to provide extrinsic signals to determine the cell survival (71, 123). Thus, outside the BM, TSNs may also be capable of maintaining LLPCs, but may be unique for specific isotypes or specificities (114–116, 118, 120, 122).

### BM Niches: Cells Involved in ASC Maintenance

The BM niche is the primary “home” for LLPCs. This ASC niche represents a highly-complex microenvironment which includes multiple cell types such as mesenchymal stromal/stem cells (MSCs) (113, 124), eosinophils (125, 126), megakaryocytes (127), basophils (128), monocytes (129), macrophages (130), dendritic cells (131), T cells (132), osteoclasts (133), and fat cells (134) (Figure 1D). Notably, the BM is physiologically hypoxic—with oxygen tensions range ~1–6%, depending upon the vicinity of blood vessels (135–138). Despite the rich cellular BM composition, it still remains a mystery whether LLPCs preferentially home to the BM or pro-actively adapt upon arrival to the extreme hypoxic BM environment.

#### MSCs (and Derivatives)

Mesenchymal stromal/stem cells (MSCs) are the principal organizer of the BM microniche involved in supporting LLPC survival. Evidence shows that this support is mediated through cell-cell contacts and/or soluble signals (62, 94–145). BM ASCs are in direct contact with MSCs (126, 146). This interaction is thought to be mediated mainly via ASC adhesion receptors, VLA-4 and LFA-1 (140, 145, 147, 148) and their natural ligands, VCAM-1 and ICAM-1, respectively, on MSCs, and are thought to anchor them to the extracellular matrix (ECM) (149). MSCs also assist ASC survival through the secretion of soluble molecules. For example, MSCs secrete CXCL12 that interacts with CXCR4 on ASCs (54, 55, 62, 150), which direct them to close proximity of MSCs. In addition to chemokines, MSCs secrete IL-6 that is critical for ASC differentiation and survival (62). Moreover, interaction of VLA-4 and fibronectin was shown to be essential for MSC-mediated survival of ASCs (148). MSCs also secrete TGF-β1 and TGF-β2, which are involved in B cell homeostasis and IgA responses in mice (139, 151–154). Finally, MSCs produce ECM-modifying molecules, such as heparan sulfate (HS) (130, 155, 156), which is an unconventional “receptor” that induces APRIL oligomerization and may trigger BCMA-mediated survival signaling of ASCs (157, 158).

The interaction between MSCs and ASCs via MSC-produced CXCL12 and ASC membrane-bound CXCR4 is well-established (159). However, it was unclear whether cell-cell contact between MSCs and ASCs was essential (126, 139, 144, 159) or dispensable (94, 150). Possible explanations for this discrepancy may be due to distinct MSC subsets for each study. Our recent work confirms that human BM-derived MSCs provided sufficient soluble factors for ASC survival without the need for cell-cell contact (94). While the persistence of BM ASCs is supported in general by MSCs, a unique subset of CXCL12^+^ MSCs are obligatory to form and maintain the survival niche (126, 146). Alternatively, the requirements for the survival of ASCs as they mature into LLPCs may vary, supporting a model that different MSC subsets are needed for distinct ASC populations (specific to blood or BM).

Recently, a unique subset of lymph node stromal cells found in the medullary cords provided a major source of ASC survival factors, including CXCL12, IL-6, APRIL, and BAFF (160). Distinct from T zone fibroblastic reticular cells (FRCs), the medullary FRC subset (MedRCs) together with macrophages promoted ASC survival by soluble signals similar to our observations with BM stromal cells. Additionally, FRCs at the GC-T cell zone interface may also have similar characteristics (160, 161). In summary, particular stromal cells in specific locations (i.e., lymph nodes, BM, or other tissue sites) may ultimately orchestrate ASC survival.

Extracellular vesicles (EVs) from BM MSCs provide additional evidence that MSC-ASC cell-cell contacts are non-essential (162). Both MSCs and ASCs are rare cell types in the BM microniche and EVs may provide survival factors over greater distances compared to the local paracrine factors which are limited by proximity. MSC-derived EVs have been shown to be involved in multiple myeloma (163), but our group recently showed that human BM MSC-derived EVs can support healthy ASC survival *in vitro* (162). Although the exact mechanism was not determined, EVs probably mediate the delivery and transfer of diverse biologically-active molecules over greater distances.

#### Eosinophils and Osteoclasts

In addition to MSCs, ASCs are also surrounded by other cell types in the BM microenvironment. Among them, eosinophils (125) and osteoclasts (OCs) (133) (Figure 1D) have been shown to promote ASC survival. Eosinophils have high-turnover, are located in the ASC vicinity, and produce survival factors, including IL-6 and APRIL (and BAFF) (125, 126). Although Chu et al. showed that eosinophils are needed for ASC survival (125), other investigators found that they are not essential (164–166). This discrepancy may be attributed to redundancy of ASC survival factors among a variety of accessory cells. Monocyte-derived OCs, which are able to support myeloma cell growth (167), also appeared to support the survival of ASCs *in vitro* (133). Interestingly, OCs produce BAFF and APRIL (168), but their ASC survival benefits may actually be independent of either APRIL or BAFF (133).

#### Other Accessory Cell Types

Other accessory cell types that are capable of promoting ASC maintenance include megakaryocytes (125, 127), basophils (125, 128), dendritic cells (131), Tfh (161, 169), and Treg (125, 170). Again, some of these cell types in BM niche may have redundant functions or secrete overlapping factors with other BM cell types and thus, may play overlapping roles for ASC survival. For example, IL-6 is paramount for ASC survival and is produced by both MSCs and eosinophils. Similarly, APRIL, another important ASC survival factor, can be secreted by neutrophils, eosinophils, megakaryocytes, osteoclasts, as well as BM MSCs (125, 127, 171). At this point, redundant functions from many BM cell types facilitate ASC persistence; however, the BM MSCs appear to be essential beyond IL-6 production for ASC survival (39, 94, 125, 126, 172). The precise soluble factors and molecular mechanisms are not known, but specific clues suggest the MSC surrounding ECM.

### BM Niches: Signals and Factors Implicated in ASC Maturation and Maintenance

In addition to via cell-cell interactions, BM niches also support the process of ASC maturation by soluble components that ultimately alter the cell phenotype into a LLPC.

#### APRIL/BAFF:BCMA and APRIL:SDC-1 Signaling

The APRIL/BAFF:BCMA signaling axis is a well-characterized pathway in ASC survival (161, 173, 174). APRIL or BAFF, two members of the TNF family of ligands, promotes ASC survival and longevity *in vitro* (125, 175, 176), in mice (123, 129, 177), and *ex vivo* (in humans) (94). These signals are primarily triggered and delivered through the interactions of BAFF/APRIL with one of their shared receptors, BCMA (176). BCMA is predominantly expressed on terminally differentiated B cells with upregulation on LLPCs (178) (Figure 2). BCMA is required for maintenance of ASCs residing within BM niches, which likely acts through APRIL (or BAFF) in combination with IL-6 (176) or CD4^+^ T cells (123). Moreover, APRIL also promotes IgA PC survival in human mucosa (116). Molecularly, APRIL mediates cell survival primarily by activation of Akt, Erk1/2, JNK, and NF-κB signaling pathways, which leads to upregulation of antiapoptotic genes. APRIL:BCMA has shown to sustain expression of Mcl-1, an important Bcl2-family antiapoptotic factor that is expressed in ASCs (85, 176).

Syndecan-1 (SDC-1), otherwise known as CD138, a heparan sulfate proteoglycan (HSPG), has been shown to be a pro-survival factor for ASCs (179) (Figure 2). While BAFF predominately binds BAFF receptor (BAFF-R; which is expressed on immature B cells), APRIL can bind through HSPGs (129, 180, 181) and thereby through SDC-1, APRIL oligomerizes and triggers BCMA mediating ASC survival. Alternatively, binding to APRIL may enable syndecans to deliver survival signals through intracellular tails (182). In humans, SDC-1 expression had been used as a LLPC surrogate (3, 42, 180); however, our group recently showed that SDC-1 can be found in early blood ASCs even though SDC-1^neg^ ASCs could eventually upregulate SDC-1 in our *in vitro* BM mimetic cultures (40). Not surprisingly, both SDC-1^+^ and SDC-1^neg^ ASCs survived similarly, concluding that upregulation of SDC-1 plays a role in ASC maintenance.

**Figure 3:**
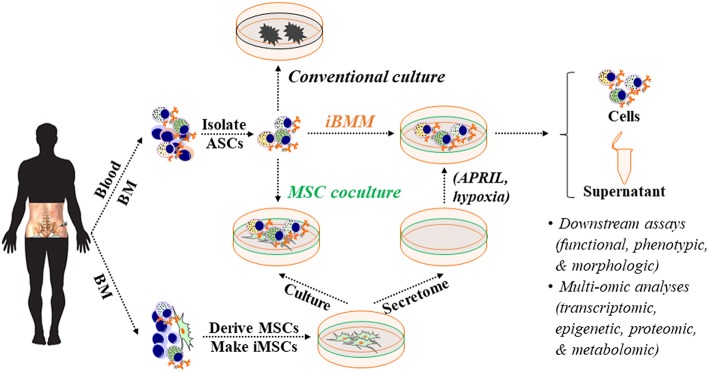
An *in vitro* BM microniche mimic (iBMM) for studying human ASCs *ex vivo*. Primary MSCs are derived from human autologous or allogeneic BM samples, expanded, and irradiated. Populations or single blood or BM ASCs are isolated and co-cultured with BM-derived iMSCs or in the cell-free secretome that is harvested from BM-derived MSCs. Typically, blood or BM ASCs quickly die off *ex vivo* or in conventional culture but survive for weeks under these conditions. The cellular materials and supernatants obtained from the *ex vivo* ASC cultures can be collected for downstream assays and multi-omic analyses for studying human ASC biology. BM, bone marrow; ASC, antibody-secreting cells; MSC, mesenchymal stromal/stem cells; iMSC, irradiated MSC; APRIL, a proliferation-inducing ligand.

**Figure 4:**
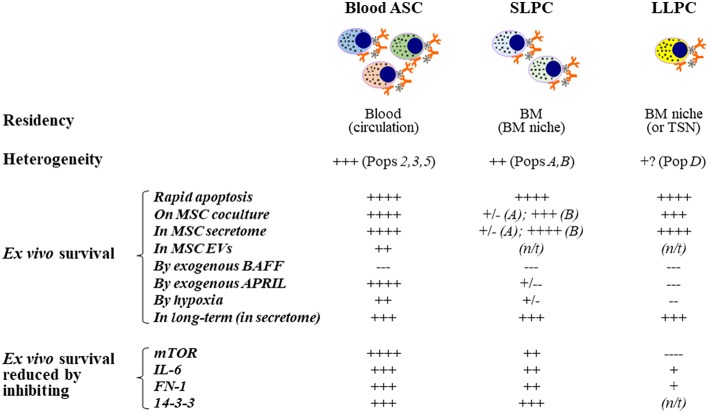
Extrinsic cues for human *ex vivo* ASC maintenance: Findings from using the *in vitro* human BM mimic. Blood ASCs circulate transiently while short- and long-lived PCs reside in tissue-specific niches like the BM. Both circulating ASC and local PC populations are heterogeneous in humans and can be classified into distinct subsets based on their expression of CD19, CD38, and CD138. These populations are named Pop2, Pop3, or Pop5 in blood and PopA, PopB, or PopD in the BM. Typically, each of these ASC populations undergo rapid apoptosis *ex vivo*; however, they can survive for several weeks to months when cultured on BM MSCs, cultured in the cell-free MSC secretome, or cultured in BM MSC-derived extracellular vesicles, albeit at a lower potency. Using this approach, we have evaluated the effect of molecules on *ex vivo* human ASC survival by adding exogenous molecules, altering the environment where the cells are cultured, and by inhibiting pathways. APRIL and hypoxia offer survival advantages to human blood ASCs but appear to have little or no effects on human BM SLPCs and LLPCs. In contrast, BAFF does not increase survival of ASCs *ex vivo* even though it is essential for B cell survival. Interestingly, inhibition of mTOR has also elucidated a dependence on this pathway for human blood ASCs but less reliance on this pathway for BM LLPCs. Other pathways have been evaluated similarly as indicated. ASC, antibody-secreting cells; PC, plasma cells; SLPC, short-lived PCs; LLPC, long-lived PCs; MSC, mesenchymal stromal/stem cells; BM, bone marrow; SLO, secondary lymphoid organs; TSN, tissue-specific niches; ex-, exogenous; EVs, extracellular vesicles; N/T, not tested or not available; long-term survival, >8 weeks.

Although there was some debate whether BAFF and APRIL equally support ASC survival (177), BAFF provides no survival advantage in our *in vitro* BM mimetic cultures (Figure 3). Only APRIL enhanced *ex vivo* survival of human early minted blood ASCs (94). Also, BAFF and APRIL do not appear to be involved in the pro-survival support to ASCs of OCs, which is reported to be fully cell-cell dependent (133). To our surprise, APRIL provided no additional enhancement over the BM MSC secretome for BM LLPCs (183) (Figure 4). Together, these data suggest that APRIL may only be needed transiently in the early blood ASC phase, permanently altering the phenotype into the LLPC maturation program.

#### IL-6:IL-6R and IL-6:IL-6ST Signaling

IL-6 signaling has been known to play a critical role in ASC differentiation and long-term survival (Figure 2). IL-6 is important for the differentiation of B cells into ASCs and acts as a pro-survival factor during ASC migration (112). Inhibiting IL-6 signaling leads to a profound reduction of human ASCs (94, 150). Early minted blood ASC subsets have a relatively high expression of the IL-6R (~32–77%), as shown in our recent study (40). Thus, during the acute phase of an infection when B cells differentiate to ASCs, when there is an abundance of free IL-6, ASCs signal through IL-6 through the cell surface IL-6 receptor (IL-6R). However, after resolution of the infection during steady state, free IL-6 levels decrease dramatically with most of it bound to the soluble IL-6 receptor (sIL-6R). The fact that, unlike blood ASCs, BM ASC have low expression of the surface IL-6R (66), might have implicated distinct mechanisms for IL-6 signaling in early minted ASCs and LLPCs.

Fortunately, IL-6 can act through two distinct signaling pathways: classic and trans-signaling. In the classic pathway, IL-6 first binds to the membrane-bound IL-6R (also known as gp80 or CD126); this interaction leads to dimerization and activation of the signal transducing protein gp130 (also known as IL-6ST or CD130), a transmembrane protein important for signal transduction following cytokine engagement through activation of the Jak-1/STAT and Akt/MAPK/ERK-1/2 signal transduction pathways, to eventually upregulate STAT3 (184–186). In the absence of IL-6R, IL-6 has no binding affinity for IL-6ST; therefore, only cells that express IL-6R can be stimulated by IL-6 (187). Thus, the effect of IL-6 through classic signaling is rather limited because it is restricted to only cell types that express the IL-6R such as early minted blood ASC (40, 188, 189). The trans-signaling activates the IL-6/STAT3 through extracellular IL-6 initially binding to its soluble IL-6R (sIL-6R), then this IL-6/sIL-6R complex subsequently binds to IL-6ST (190). Through trans-signaling, IL-6 (and the sIL-6R) stimulates cells that lack the membrane-bound IL-6R. Interestingly, IL-6ST is virtually expressed on all cells in the body (191), including BM LLPCs. It will be interesting to corroborate evidence from the transcriptomes of early minted ASCs and BM LLPCs for IL-6ST expression and investigate whether IL-6 trans-signaling plays a role in LLPC survival.

#### CXCL12:CXCR4 Signaling

As aforementioned, CXCR4 (CD184) and its ligand CXCL12 (SDF-1) play an essential role in directing blood ASCs to BM (42, 54, 55). Whether CXCL12 can directly also act as an ASC survival factor *in vivo* (192), or merely functions as a retention factor to maintain ASCs in survival niches is not known (193). One study showed that CXCL12:CXCR4 signaling promotes ASC survival *in vitro* (62). Nonetheless, further studies are needed to discriminate survival vs. homing mechanisms of CXCL12.

#### Other Soluble and Membrane-Bound Survival Factors

Additional survival factors reported to date include TGF-β1 (as mentioned previously) (139, 153, 154), IGF-1, TNF-a, HGF, VEGF, IL-21 (125, 130, 159, 161, 173), and fibronectin-1 (FN-1) and YWHAZ (14-3-3 zeta/delta) (94, 148) (Figure 2). Among some of the cell surface molecules involved in ASC survival are CD28 (131, 194, 195), CD93 (196), CD44 (62), CD37 (197), FcgRIIb (198), LFA-1, CD49d (VLA-4), CD49e (VLA-5) (130, 142, 148), CD73 (199), CD13 (YWHAZ receptor) (200, 201), and CD123 (IL-3R) (202). Although some of the factors, such as CD28 (131, 195) and CD93 (196), have been shown to be important for the Ab secretion and survival of BM ASCs, the other factors will probably need further evaluation to understand if they are essential or redundant factors for healthy LLPC maturation and maintenance.

## Novel BM Mimetic Supports ASC Survival and Maturation *EX VIVO*

Mouse and human ASCs undergo rapid apoptosis *ex vivo* (62, 94, 142, 162). Therefore, studies to understand human ASC survival *in vitro* have not been possible. To overcome this issue, we developed a novel culture system that mimics the human BM microniche to support ASC survival *ex vivo* to interrogate mechanisms of LLPC maturation (94). To establish this system, we derived and expanded primary human BM MSCs, which are spindle-shaped, plastic-adherent, and phenotyped as CD90^+^CD73^+^CD45^−^CD19^−^ (94) (Figure 3). We initially utilized irradiated BM MSCs as feeders for *in vitro* cultures of ASCs but quickly found their pro-survival support was entirely driven through paracrine effects, thus defining cell-free BM-derived MSC secretome (94). In contrast, we did not find prolonged ASC survival from cell types found in the blood (94).

We also found that the BM MSC secretome alone was not sufficient for long-lived ASC survival and exogenous APRIL and BM hypoxic conditions provided enhanced longevity (Figures 3, 4). We refer to the BM MSC secretome, exogenous APRIL, and hypoxic conditions as the human cell-free *in vitro* BM mimetic (iBMM). Previous systems supported human ASCs with co-cultures of supportive cells for a few days or from *in vitro* generated ASCs (139, 175, 203). Our unique system maintains freshly isolated human ASCs *ex vivo* for 8–12 weeks and beyond (94).

The development of iBMM has been critical for studying human ASCs *in vitro*. It provides powerful tools to follow the human ASC maturation process in the BM microniche to characterize the survival factors, signaling pathways, or metabolic programs important for LLPC generation. Moreover, by coupling recent advances in single cell molecular analysis, epigenetics, proteomics, and metabolomics together with the *in vitro* iBMM, we will be able to dissect the mechanisms of LLPC generation and provide novel insights to their survival and modulation of Ig secretion.

Additionally, the iBMM or cell-free ASC survival system provides a rapid novel system for monoclonal Ab discovery. Currently, monoclonal antibody discovery is limited by cost and time, limiting the ability to identify rare clones. With this system, a priori selection of rare but targeted ASC clones can be identified prior to monoclonal Ab generation. For example, Abs for only neutralizing epitopes can be selected for monoclonal Ab generation, thereby decreasing cost and time.

The ability to support human blood and BM ASC survival *ex vivo* for months may also provide novel assays to measure vaccine durability. Most vaccine candidates go through extensive clinical testing to assess immunogenicity, safety, and efficacy, and at this time, assessment of vaccine longevity requires the tincture of time. By using the BM mimetic, antigen specificity and neutralization capacity (i.e., protectiveness), of the Ab secreted from circulating ASCs can easily be assessed prior to serum level. Additionally, durability of the vaccines may also be assessed *in vitro* using the iBMM.

In summary, the generation of LLPCs is complex and involves both intrinsic and extrinsic factors. B cell differentiation to an ASC is immensely intricate involving GC and non-GC reactions together with upregulating the ASC programs. Although ASC differentiation is necessary it is not sufficient for LLPC generation, which involves BM survival factors to maintain ASC Ab secretion as well as other extrinsic elements in the microniche that appear to fundamentally transform early minted ASCs into a LLPC phenotypes. The novel iBMM culture system provides tools for studying LLPC generation and maintenance. Coupling with the recent advances in transcriptomics, at both a high-throughput scale and single-cell resolution, this system has the potential to answer questions about the intrinsic and extrinsic factors that regulate differentiation, maturation, and longevity of human LLPCs. Despite the progress in understanding the extrinsic cues that support ASC survival and Ab secretion, more studies are needed to characterize the complex maturation and maintenance of LLPCs.

## Author Contributions

DN, CJ, and FL wrote the manuscript. IS provided important editorial input.

### Conflict of Interest Statement

FL founded MicroB-plex, Inc. The remaining authors declare that the research was conducted in the absence of any commercial or financial relationships that could be construed as a potential conflict of interest.
